# Coronary Steal Syndrome Secondary to Large Coronary to Pulmonary Artery Fistulas

**DOI:** 10.7759/cureus.30267

**Published:** 2022-10-13

**Authors:** Christian Torres, Medeona Gjergjindreaj, Hernando Torres-Ortiz, Jorge Fuentes, Nirat Beohar

**Affiliations:** 1 Cardiology, Columbia University Division of Cardiology at Mount Sinai Medical Center, Miami Beach, USA; 2 Interventional Cardiology, Columbia University Division of Cardiology at Mount Sinai Medical Center, Miami Beach, USA

**Keywords:** coronary steal phenomenon, coronary artery steal syndrome, transarterial coil embolization, right coronary artery to pulmonary artery fistula, coronary to pulmonary artery fistula, left anterior descending coronary artery to pulmonary artery fistula, coronary artery fistula (caf)

## Abstract

Coronary artery fistulas represent rare congenital or acquired defects in the coronary circulation. We describe a case of bilateral coronary to pulmonary artery fistulas resulting in coronary artery steal syndrome in a patient with a history of valve-sparing aortic repair surgery.

## Introduction

Although a rare entity, coronary artery fistulas (CAFs) can present with various signs and symptoms depending on the number, size, location, hemodynamic profile, and underlying patient comorbidities [1]. When large, CAFs can induce sequestration of blood toward a low-resistance cavity like the pulmonary artery resulting in coronary steal syndrome [2]. We report a case of a 50-year-old male who presented with complaints of chest pain, dyspnea on exertion, and a decline in functional status secondary to dual CAFs toward the pulmonary artery with a successful percutaneous embolization.

## Case presentation

History of presentation

A 50-year-old male with a past medical history significant for aortic root aneurysm repair one year prior to presentation was evaluated for palpitations, chest pain, dyspnea on exertion, and progressive decline in functional status since his surgery. Coronary CT showed multiple communications between the coronary and pulmonary artery (PA) circulation. A coronary angiogram confirmed the presence of coronary to pulmonary artery fistulas (CPAFs) (Videos 1, 2). Subsequent right heart catheterization (RHC) showed significant shunting of coronary blood flow into the pulmonary circulation. The patient underwent successful percutaneous embolization of the largest fistula originating from the ostium of the right coronary artery (RCA).

**Video 1:** Coronary angiogram anteroposterior (AP) cranial view Anteroposterior cranial view showing the right coronary system giving origin to a large aneurysmal coronary to pulmonary artery fistula and a small RCA. PA: pulmonary artery; RCA: right coronary artery.

**Video 2:** Coronary angiogram right anterior oblique (RAO) caudal view RAO caudal view showing the left coronary system composed of the LAD and the LCX. The LAD gives origin to multiple abnormal coronary artery fistulas anastomosing into the PA. LAD: left anterior descending artery; LCX: left circumflex artery; PA: pulmonary artery.

Past medical history

Medical history was notable for hypothyroidism treated with levothyroxine, as well as essential hypertension treated with diltiazem, atenolol, and enalapril.

Past surgical history

The patient was diagnosed with an aortic root aneurysm at the age of 49, for which he underwent valve-sparing aortic root replacement using a 30 mm Maquet Cardioroot (Rastatt, Germany). Notably, a preoperative coronary angiogram showed no significant obstructive coronary artery disease (CAD) or CAFs.

Differential diagnosis

Valvular heart disease, congestive heart failure, pulmonary hypertension, and aneurysm recurrence were in the initial differential diagnosis, given the patient’s subacute presentation of exertional dyspnea and progressive functional decline following aortic root replacement.

Investigations

Vital signs on presentation were remarkable for hypertension. The physical exam was largely unremarkable, as was the laboratory workup, including a normal basic metabolic profile, complete blood count, troponin, and N-terminal pro-B-type natriuretic peptide (NT-pro-BNP). Electrocardiogram was also unrevealing with no significant ST segment or T wave changes.

Transthoracic echocardiogram (TTE) demonstrated a normal ejection fraction with a normal trileaflet aortic valve with mild aortic regurgitation and a normal-appearing aortic root and ascending aorta with a Cardioroot graft in the proximal segment. CT angiography showed no evidence of pulmonary embolism or significant CAD; however, on further review, multiple moderate-sized vessels branching from the right and left coronaries were demonstrated anastomosing to the pulmonary trunk suggestive of left to right shunt from a CPAF.

**Figure 1:**
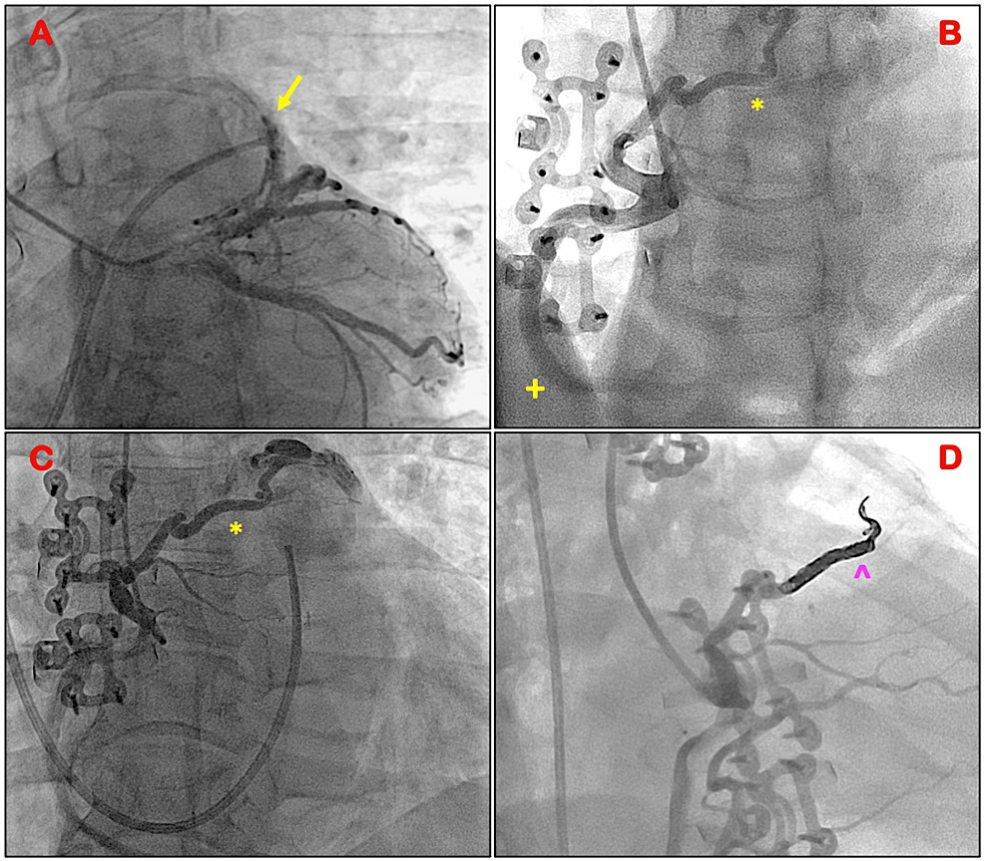
Coronary angiogram A: LAO caudal view showing the left coronary system. The yellow arrow demonstrates multiple small to moderate-sized collateral originating proximally from the LAD anastomosing to the main PA. B & C: LAO cranial view of the RCA demonstrating the true RCA. * highlights the large anomalous artery originating at the ostium of the RCA coursing superiorly and anteriorly anastomosing into the main PA distally. D: ^ Deployment of two Penumbra Ruby coils (3 mm x 20 cm and 3 mm x 15 cm) with no residual flow distally. LAO: left anterior oblique; LAD: left anterior descending artery; PA: pulmonary artery; RCA: right coronary artery.

A subsequent coronary angiogram was performed, excluding the presence of significant CAD. Incidentally, the left anterior descending artery (LAD) was noted to give rise to multiple small to moderate-sized vessels branching from the proximal and mid-LAD anastomosing to the main PA (Figure 1A). The left circumflex artery (LCX) also had small vessels branching lateral to the LCX, anastomosing to the main PA distally. The RCA branched into two large-caliber vessels at the ostium. The true RCA had mild luminal irregularities. The second branch was a larger anomalous artery that originated at the ostium of the RCA and coursed superiorly and anteriorly anastomosing into the main PA distally (Figures 1B, 1C and Videos 1, 2).

An RHC was also performed, revealing normal right-sided pressures with significant oxygen step-up between the right atrium (RA) and the PA consistent with a hemodynamically significant shunt (RA: 70%; PA: 80%).

Management

Given the patient’s presentation, as well as the anatomic and hemodynamic findings, the decision was made to proceed with coil embolization of the large RCA to PA fistula.

Using a micropuncture technique, a 6-F sheath was inserted into the right common femoral artery. A 6-F AL1 guide catheter was used to selectively engage the origin of the fistula. A Renegade microcatheter (Boston Scientific, Marlborough, MA) was successfully advanced into a satisfactory position over a guidewire, followed by the deployment of two Penumbra Ruby coils (3mm x 20cm and 3mm x 15cm; Penumbra, Inc., Alameda, CA) with no residual flow distally and no angiographic complications (Figure 1D). The residual CAFs in the LAD and LCX were considered not to be large enough to require an intervention. The femoral access was closed using a 6-F Angioseal (Terumo Corporation, Somerset, NJ). The patient was discharged home 48 hours following the procedure with excellent recovery and symptomatic improvement.

The patient had an uneventful peri-procedural recovery and was discharged home 48 hours post-procedure.

Follow-up

He had an excellent recovery and symptomatic improvement prior to discharge. The patient continued to do well at a six-month follow-up with no recurrence of symptoms.

## Discussion

CAFs are rare, the majority of which are congenital, accounting for only 0.9% of congenital heart anomalies [3]. Acquired CAFs have been described following open-heart surgery, especially when revascularization is performed and after radiation therapy [4,5]. The prevalence of CAFs has increased over the last few years, often incidentally found due to the increasing availability of CT angiography [3]. Although frequently asymptomatic, CAFs can create significant alterations in the coronary circulation, causing myocardial ischemia by sequestration and shunting of oxygenated blood away from myocardial tissue resulting in a coronary steal syndrome physiology with subsequent long-term complications like heart failure and arrhythmias [4]. Moreover, pulmonary hypertension has also been described as a result of blood shunting to the pulmonary vasculature [6].

CAFs more commonly drain into the right ventricle and right atrium, with only 17% of them reported draining into the pulmonary circulation [7]. CPAFs account for 15-30% of CAFs and can originate from any coronary territory. Although initially thought to have a predilection for the RCA [8,9], a recent systematic review identified the left main/LAD (84%) to be the most common coronary artery of origin for a CPAF, followed by the RCA (38%) [10]. Our patient had multiple bilateral CAFs, the largest originating at the ostium of the RCA and anatomized into the main PA. After a multidisciplinary discussion, the decision was made to attempt a percutaneous embolization of the RCA CPAF thought to be the predominant source of his symptoms based on RHC hemodynamic assessment showing a significant step up in oxygen saturation.

To date, there are no dedicated guidelines regarding the management of CAFs [11]. Several authors advocate for the closure of symptomatic patients, the presence of end-organ damage, or those with a high-risk hemodynamic profile. Although surgical intervention has been shown to have low morbidity, it is associated with a higher risk of complications. Additionally, as in other cardiovascular conditions, many patients are deemed high or at a prohibitive surgical risk [12]. Multiple successful case reports, including ours, have shown percutaneous closure of such anomalies to be a safe and effective technique [13,14].

## Conclusions

CPAFs are rare, most of them congenital; however, acquired fistulas following cardiac surgery have been described. Commonly asymptomatic and incidental, CPAFs carry a potential risk of compromising coronary circulation resulting in myocardial ischemia, left ventricular dysfunction, and arrhythmias. Multimodality imaging is essential in the evaluation and management of CPAF. The use of a percutaneous coil embolization (PCE) showed an excellent result in our patient, similar to reports from all over the world. PCE is a minimally invasive and safe alternative for the management of CPAFs. It not only avoids an open sternotomy but a higher risk of complications has been reported with surgery. The tortuosity of these anomalous vessels can pose a challenge when using a percutaneous approach requiring extra support and stiffer wires.
